# Quantifying forearm and wrist joint power during unconstrained movements in healthy individuals

**DOI:** 10.1186/1743-0003-11-157

**Published:** 2014-11-17

**Authors:** Diana Castillo Flores, Simon Laurendeau, Normand Teasdale, Martin Simoneau

**Affiliations:** Département de kinésiologie, Université Laval, Quebec City, Canada; Centre de recherche du CHU de Québec, Québec, Québec, Canada; Ingeniería Biomédica, Universidad Iberoamericana, Mexico City, Mexico

## Abstract

**Background:**

Wrist movement-related injuries account for a large number of repetitive motion injuries. Remarkably little, if any, empirical data exist to quantify the impact of neuromuscular disorders affecting the wrist or to validate the effectiveness of rehabilitation training programs on wrist functions. The aim of this project was to develop a biomechanical model for quantifying wrist and forearm kinetics during unconstrained movements, to assess its reliability and to determine its sensitivity.

**Methods:**

Twenty healthy subjects with no history of upper arm and wrist pain volunteered for the experiment. To evaluate the reliability of the data, we quantified their forearm and wrist kinetics on two different days (minimum and maximum number of days between experimental sessions were 1 and 4 days respectively). To measure forearm and wrist kinetics, an apparatus was built to offer rotational inertia during forearm and wrist movements. An inertial measurement unit was located near the top of the device measuring its angular position along the frontal and sagittal planes. We used a mathematical model to infer forearm and wrist torque. Thereafter, we calculated the product of torque and angular velocity to determine forearm and wrist power.

**Results:**

Results revealed that for 75% of the power and torque measurements the ICC was greater than 0.75 (range: 0.77 – 0.83). Torque and power measurements for adduction movements, however, were less reliable (i.e., ICC of 0.60 and 0.47, respectively) across testing sessions. The biomechanical model was robust to small measurement errors, and the power peaks between the first and second testing session were not different indicating that there was no systematic bias (i.e., motor performance improvement) between testing sessions.

**Conclusions:**

The biomechanical model can be used to assess the effectiveness of rehabilitation programs, document the progression of athletes or conduct research-oriented testing of maximum forearm and wrist kinetic capacities. Nonetheless, caution should be taken when assessing forearm and wrist power adduction movements. Future studies should aim at defining a set of normative values, for various age groups, for forearm and wrist joint torque and power in healthy individuals.

**Electronic supplementary material:**

The online version of this article (doi:10.1186/1743-0003-11-157) contains supplementary material, which is available to authorized users.

## Background

Measurement of upper extremity kinematics and kinetics is a requirement in the field of neurorehabilitation, ergonomics and sports performance. For applications involving these measurements, the use of lightweight microelectromechanical systems (MEMS) inertial sensors can be useful [1, 2]. These sensors have been used to assess the efficiency of human movement such as upper arm reaching movements [3, 4]. Remarkably, there is a scarcity of information available for wrist and forearm control, even though these segments represent important components of upper limb function. Repetitive motions, large forces or any exposure to extreme positions are important risk factors in the development of wrist injuries [5]. Wrist injuries are associated with different activities in daily living. For example, when rock climbing, a series of repetitive high finger grip forces and torque movements of the upper limbs are needed to ascend a rock face creating large internal forces, which potentially result in ligament and tendon sprains or rupture [6]. The incidence of wrist injury is also high in professional and low-handicap golfers because of repetitive ulnar and radiation deviation, and the force transmitted to the wrist and hand when the club hits the ball and ground [7]. Furthermore, individuals with upper extremity musculoskeletal disorders that engage in repetitive work (computer users, factory workers and musicians) often have limited wrist range of motion (ROM), which has been attributed to increased antagonist muscle tension [8]. Increased antagonist muscle tension in the upper limb was inferred from limitations in wrist ROM and may be a source of biomechanical stress during both occupational and non-occupational activities [9].

Forearm rotation is a complex motion of the radius around the ulna. Although the range of motion is typically used to assess elbow, forearm and wrist performance, it is likely that forearm and wrist kinetic capacities play a crucial role during daily activities. Regarding clinical applicability, although the link between forearm and wrist torque or power and forearm and wrist joint disorders is unclear, it is likely that individuals with impaired forearm and wrist joint strength who repetitively execute movements to move heavy objects would experience forearm and wrist control instability leading to pathologies. Wrist injuries cause a significant amount of lost work time for both male and female workers. According to data from the United States Bureau of Labor Statistics (more than 465 000 nonfatal occupational injuries are included), for US industries in 1995, approximately 30% of wrist injuries required 31 or more days away from work, and only 10% of those sustaining wrist injuries returned to work after only one day off [10]. According to these numbers, there is a need for the development of optimal training programs for improving wrist joint recovery time. Athletes, musicians and workers would benefit if a valid and reliable biomechanical model and device existed to quantify forearm and wrist kinetics during unconstrained movements. This could be useful for tracking improvement during training programs or to document the progression of a rehabilitation program aiming at maximizing forearm and wrist muscle recovery following injury. The main objectives of this study were threefold: i) to develop a biomechanical model quantifying forearm and wrist kinetics during unconstrained 3D movements, ii) to assess the reliability of the biomechanical model and iii) to quantify the robustness of the biomechanical model.

## Methods

**Figure 1:**
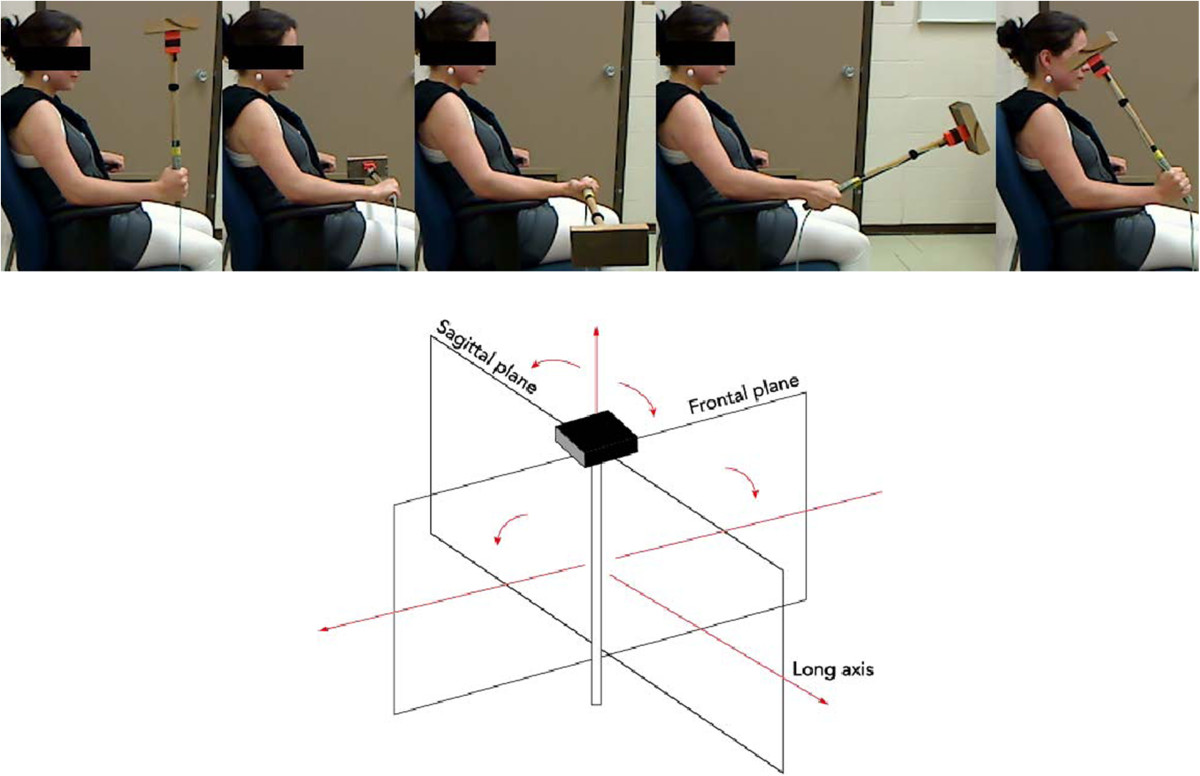
**Experimental conditions.** Upper panel) The participant’s starting position and final position for pronation, supination, adduction and abduction experimental conditions. Lower panel) Representation of the inertial device as a rigid inverted pendulum rotating around the long axis of the forearm, for pronation/supination movements (primary plane: frontal), and along an axis perpendicular to the long axis of the forearm, for adduction/abduction movements (primary plane: sagittal). For illustration purposes, the planes are rotated.

Twenty healthy participants (10 males and 10 females; mean age 25.5 ± 5; mean height 1.76 m; mean weight 72.5 kg) with no history of upper arm and wrist pain volunteered for the experiment. Fifteen participants reported they were right hand dominant and five participants reported they were left hand dominant. To evaluate the reliability of the data, we quantified their wrist and forearm kinetics on two different days (minimum and maximum number of days between experimental sessions were 1 and 4 days respectively). Each participant signed the informed consent form outlining the protocol approved by the Laval University Institutional Review Board.The participants performed four different wrist movements: i) pronation, ii) supination, iii) adduction and iv) abduction (Figure 1). They performed 12 trials per condition. Considerable care was taken to minimize fatigue, and participants rested for 2 minutes every 6 trials or when they asked for a break. The presentation order of the experimental conditions was always the same: pronation, supination, adduction and abduction, and all movements were performed with the right forearm. Participants were seated comfortably with their back resting on the back of a chair and with their right forearm resting on the chair armrest, which reduced elbow translation and limited shoulder motion. At the beginning of each trial, participants were asked to align the inertial device with the vertical axis, and the angle between the right forearm and the inertial device was approximately 90°. They were instructed to move the inertial device as fast as they could and to maintain the final position until they were told to move back to the initial position. In addition, they were instructed to produce movements along a single plane, that is, along the frontal plane or along the sagittal plane, respectively for pronation/supination and adduction/abduction movements.

To measure forearm and wrist kinetics, an apparatus was built to offer rotational inertia during movements. The inertial device was made of wood and had a hammer-like shape (Figure 1). An inertial measurement unit (Xsens Technologies, model MTx, Enschede, Netherlands) was located near the top of the device measuring its angular position along the frontal and sagittal planes. In the initial position, the angular orientation along the frontal and sagittal planes was approximately 0°. The signals of the inertial motion unit were sampled at 100 Hz with 16-bit resolution. Angular positions were filtered using a zero lag low-pass Butterworth filter (cut-off frequency 6 Hz using a fourth order). The angular position along each plane was differentiated numerically with a central finite difference technique to obtain the angular velocity and the angular acceleration [11].

We experimentally assessed the moment of inertia of the inertial device along both planes. To do so, we had the inertial device acting as a pendulum and recorded its angular position across time using the same inertial motion unit. In the initial position, the orientation of the inertial device was horizontal. Following the release, the inertial device swung back and forth and eventually stopped when the kinetic energy was too small to produce any movement. Ten trials along both planes were acquired. We used mathematical modeling to infer the moment of inertia of the inertial device which was modeled as a pendulum rotating around a pivot. The model was implemented using Matlab and Simulink toolbox (Mathworks, Natick, MA, USA). We used the Optimization toolbox (lsqnonlin function) to iteratively adjust the moment of inertia (J) by minimizing an error function (i.e., the vector differences between the predicted angular position and the measured angular position). We determined the goodness-of-fit by calculating the variance accounted for by the model. Overall, the model explained 93.5% and 96.8% of the variance in the experimental data for the frontal and sagittal planes, respectively. The moment of inertia of the inertial device along the frontal and sagittal planes were 0.049 kgm^2^ and 0.046 kgm^2^, respectively.

Subsequently, we used a mathematical model to infer forearm and wrist torques along the frontal and sagittal planes (Eq. 1). We modeled the inertial device as a rigid inverted pendulum rotating around the long axis of the forearm (coplanar with the sagittal plane) and along an axis perpendicular to the long axis of the forearm (coplanar with the frontal plane). Because the participant’s forearm rested on the chair armrest, we assumed that movement of the axes of rotation was negligible.

1 $$\begin{array}{ll} \left[\begin{array}{c} {\vec{\tau }}_{F}\\ {\vec{\tau }}_{S} \end{array}\right]= & \left[\begin{array}{ll} J_{XX} & J_{XY}\\ J_{YX} & J_{YY} \end{array}\right]\times \left[\begin{array}{l} {\vec{\overset{\cdot \cdot }{\theta }}}_{1}\\ {\vec{\overset{\cdot \cdot }{\theta }}}_{2} \end{array}\right]+M\times g\\ \times L\left(\left[\begin{array}{c} 1\\ 0 \end{array}\right]\times \sin\left({\vec{\theta }}_{1}\right)+\left[\begin{array}{c} 0\\ 1 \end{array}\right]\times \left(\cos\left({\vec{\theta }}_{2}\right)\times \sin\left({\vec{\theta }}_{2}\right)\right)\right) \end{array}$$

In this equation, ${\vec{\tau }}_{F}$ and ${\vec{\tau }}_{s}$ represent the forearm and wrist torques along the frontal and sagittal plane. J is a 2 × 2 diagonal matrix (*J*_*XX*_ and *J*_*YY*_) representing the moment of inertia of the inertial device about the axis of rotation; $\vec{{\ddot{\theta }}_{1}}$ and $\vec{{\ddot{\theta }}_{2}}$ are the angular accelerations along the frontal and sagittal planes; M is the mass of the inertial device (M = 0.34 kg); g is the gravitational acceleration (g = 9.81 m/s^2^); L is the distance between the center of rotation (0.06 m from the end of the inertial device) and the center of mass of the inertial device (L = 0.34 m); and $\vec{\theta_{1}}$ and $\vec{\theta_{2}}$ are the angular positions of the inertial device along the frontal and sagittal planes, respectively.

From the time series, we computed the power along both planes using the inner product of forearm and wrist joint torques and the angular velocities (Eq. 2).

2 $$\left[\begin{array}{c} P_{F}\\ P_{S} \end{array}\right]=\left[\begin{array}{c} {\vec{\tau }}_{F}\\ {\vec{\tau }}_{S} \end{array}\right]•\left[\begin{array}{c} {\vec{\dot{\theta }}}_{1}\\ {\vec{\dot{\theta }}}_{2} \end{array}\right]$$

### Reliability analysis

Test-retest reliability can be calculated from measurements of the same participants on two occasions using the intra-class correlation coefficient (ICC). We used Shrout and Fleiss’s [12] ICC(3,k) model. Therefore, for each participant, mean power per condition for session 1 and session 2 were considered. As mentioned above, participants were instructed to produce movements along a single plane: a) pronation/supination movements along the frontal plane, and b) adduction/abduction movements along the sagittal plane. Movements were not constrained physically and some movement along the other plane could be observed but all trials were kept and analyzed. Reliability values along the primary plane (i.e., frontal for pronation/supination movement and sagittal for adduction/abduction movements), however, are reported. ICC values are considered to reflect the following: a poor reliability when below 0.40; a fair to good reliability from 0.4 to 0.75; and an excellent reliability when greater than 0.75 [13]. The standard error of measurement (SEM) is not affected by inter-subject variability and is important for clinical utilization of a measurement procedure. The SEM is indicative of the range of scores that can be expected on the retest. It was calculated for forearm and wrist joint torque and power along the primary plane. Finally, for forearm and wrist torque and power, 2-tailed paired t-tests between the test and retest scores were calculated to verify if participants systematically improved their performance across testing sessions (i.e., systematic bias).

### Sensitivity analysis

Biomechanical models are prone to measurement errors. Consequently, to predict the effect of slight measurement errors on peak power values, the length, the mass and the moment of inertia of the inertial device were altered by ±5% (by a step size of 1%) of their measured values. Each change in the parameters was made independently of the others; therefore, interaction effects were not calculated. From the experimental data of all participants recorded during session 1, measurement errors were added to the experimental time series (i.e., angular kinematics) to simulate forearm and wrist power. From these data, for each participant, means of peak power along both planes and for each simulated condition were calculated. Mean values for each condition are reported.

## Results

**Figure 2:**
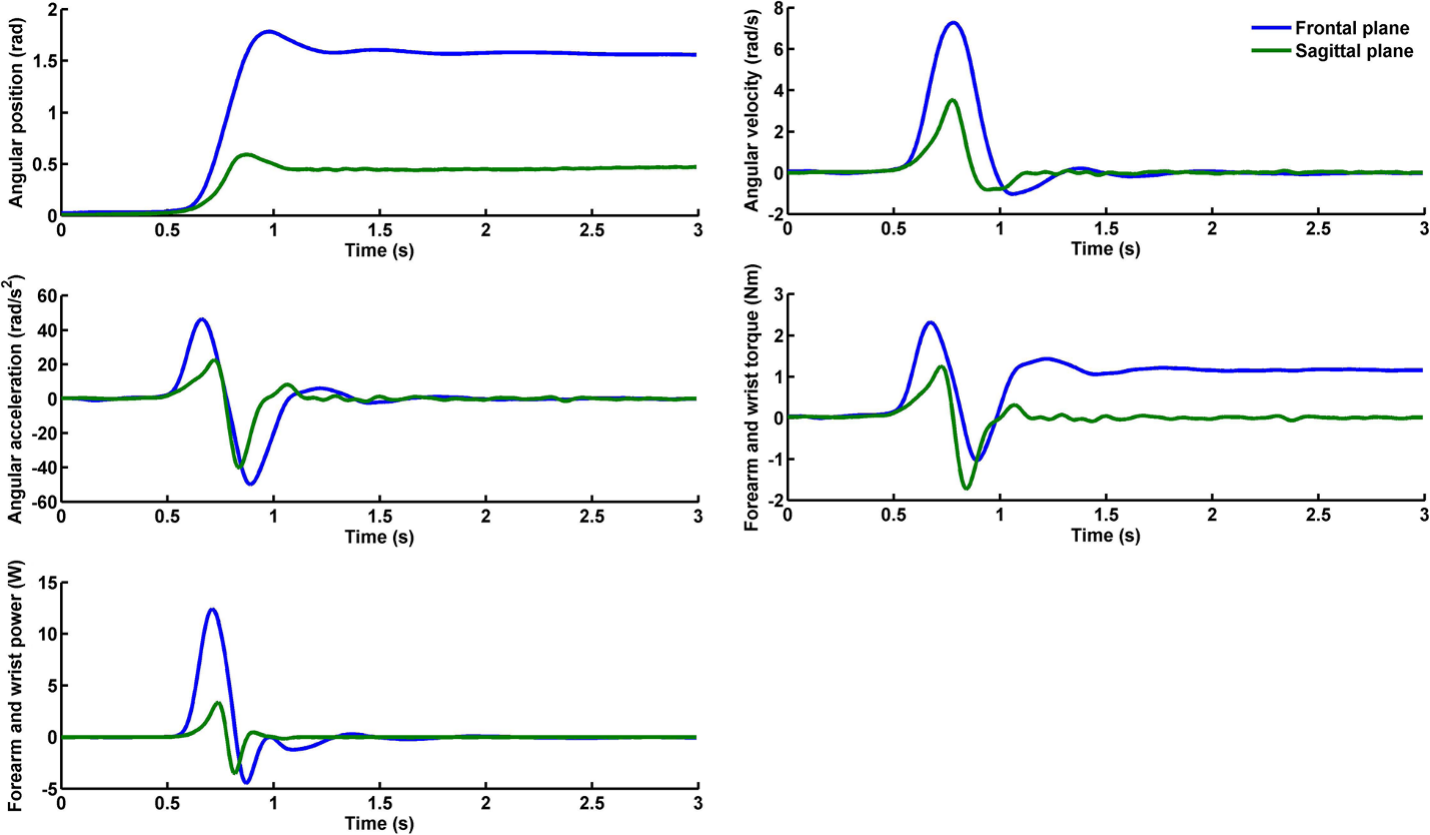
**Examples of experimental time series.** Typical time records of the kinematics and kinetic variables for the pronation condition. Upper left panel) Angular position of the inertial device. Upper right panel) Angular velocity of the inertial device. Left middle panel) Angular acceleration of the inertial device. Right middle panel) Forearm and wrist torque. Bottom left panel) Forearm and wrist power. Data along the frontal plane (blue lines) and the sagittal plane (green lines) are illustrated.

For each trial, forearm and wrist torque and power were calculated from the kinematics of the inertial device (Figure 2). Although participants were instructed to perform a specific motor action (e.g., pronation), because they were not physically constrained, movements along the other plane were observed. For each trial, the first peak torque (negative or positive depending on the motor action) and the first positive peak power were extracted; these data are related to the maximum concentric work and were used to perform the reliability analysis.

**Figure 3:**
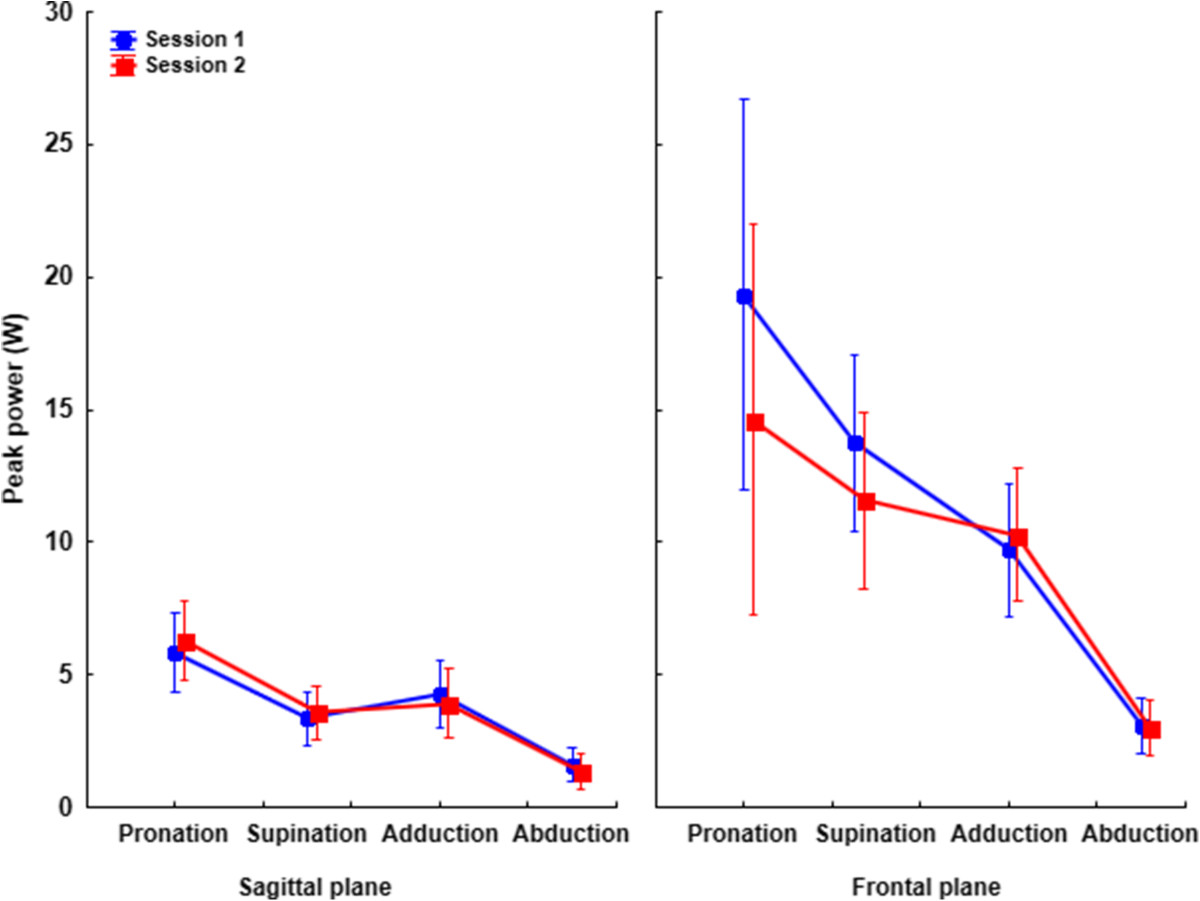
**Mean kinetics data.** Mean (±95% confidence interval) forearm and wrist power for all experimental conditions along the sagittal plane (left panel) and frontal plane (right panel) for session 1 (blue lines) and session 2 (red lines).

The reliability analysis (Table 1) revealed excellent reliability for 75% of the power and torque measurements (i.e., 6 of 8 measurements) since the ICC was greater than 0.75 (range: 0.77 – 0.91). Nonetheless, fair to good reliability was observed for torque and power in adduction as the ICC values were respectively 0.60 and 0.47. Compared to the other motor actions, the SEM was slightly larger for wrist power during pronation and supination (Table 1). Nonetheless, the values represent respectively 19.10% and 13.11% of the average data. The comparison of peak power between the first and second testing sessions (2-tailed paired t-test) revealed no difference (Figure 3). This result indicates that there was no bias between testing sessions (Table 2).

**Table 1:** **Intra-class correlation values**

Measurements	ICC	95% ICC	SEM	MP
**Frontal plane**				
**Torque (Nm)**				
Pronation	0.82	0.54-0.93	0.22	2.53
Supination	0.80	0.47-0.92	0.15	-2.19
**Power (W)**				
Pronation	0.77	0.43-0.91	3.11	16.96
Supination	0.79	0.47-0.92	1.46	12.66
**Sagittal plane**				
**Torque (Nm)**				
Adduction	0.60	0.00-0.84	0.11	-1.34
Abduction	0.83	0.57-0.93	0.09	-0.44
**Power (W)**				
Adduction	0.47	0.00-0.77	0.64	4.10
Abduction	0.79	0.46-0.92	0.32	1.47

Intra-class correlation values (ICC), standard errors of the mean (SEM) and mean peaks (MP) for wrist joint torque and power for all experimental conditions.

**Table 2:** **T-test between testing sessions**

	Power	Torque
**Frontal plane**		
**Pronation**	T_19_ = 1.47, p = 0.16	T_19_ = 1.43, p = 0.17
**Supination**	T_19_ = 1.47, p = 0.16	T_19_ = -1.40, p = 0.18
**Sagittal plane**		
**Adduction**	T_19_ = 0.42, p = 0.68	T_19_ = -0.18, p = 0.86
**Abduction**	T_19_ = 0.98, p =0.34	T_19_ = -0.06, p = 0.95

Results of the paired t-test between session 1 and session 2 for forearm and wrist power and torque along primary plane.

For the sensitivity analysis, the maximal changes in peak forearm and wrist joint power were compared with the standard error of the mean (Table 3). The standard errors of the mean in peak forearm and wrist power were larger than the increases in peak power due to simulated measurement errors, regardless of the motor action (e.g., pronation or supination) and plane (frontal or sagittal). This result suggests that the biomechanical model is robust to slight measurement errors in the moment of inertia, mass or the distance between the axis of rotation and the center of mass of the inertial device.

**Table 3:** **Sensitivity analysis**

	Simulated measurement errors		Experimental data
	**Error in moment of inertia (J)**	**Error in length (L) or mass (M)**	**Standard error of the mean (SEM)**
**Frontal plane**			
**Pronation**	0.79 ± 0.24	0.19 ± 0.01	3.11
**Supination**	0.24 ± 0.04	0.05 ± 0.004	1.66
**Sagittal plane**			
**Adduction**	0.13 ± 0.03	0.03 ± 0.008	0.45
**Abduction**	0.07 ± 0.02	0.004 ± 0.001	0.23

Change (mean ± SEM) in forearm and wrist power due to simulated measurement errors.

## Discussion

Although data exist concerning wrist isometric strength capabilities [14, 15] or range of movement [16], to our knowledge, there is no device permitting the quantification of wrist joint torque and power during unconstrained 3D movements. Unfortunately, isometric strength testing does not assess the ability of the wrist joint to control complex movements [17, 18]. An important component of the wrist and forearm is the ability to activate the appropriate muscles either to accelerate/decelerate the hand or to resist external forces in any directions (e.g., moving a hammer). Therefore, the aims were to develop a biomechanical model to quantify wrist joint kinetics while participants performed unconstrained movements, to assess the model reliability and to determine whether the biomechanical model was robust to small measurement errors.

Overall, the results suggest that the biomechanical model is reliable and robust to small measurement errors. For 6 out of 8 power and torque measurements, the ICC values were greater than 0.75 representing excellent reliability. The ICC values for torque and power during adduction movements were lower (i.e., 0.60 and 0.47, respectively). We do not have a clear-cut explanation for this result; however, we noticed that during wrist adduction, the axis of rotation of the inertial device moved slightly more than during pronation or supination movements. As a result, adding the 3D kinematics of the axis of rotation in the biomechanical model could improve the reliability for adduction movements. Nonetheless, a sensor should be added to measure the kinematics of the axis of rotation; this would increase the cost and the data processing time (i.e., data from both sensors would need to be synchronized), and it would require a more complex biomechanical model. Another possibility is that participants were fatigued despite the fact that various rest periods were taken. This suggestion is unlikely because the abduction condition was performed last and the reliability for torque and power were 0.83 and 0.79, respectively. The most difficult challenge in reliability testing is when the response being measured is inherently unstable. It is possible that during adduction movements, ligaments were stretched to a greater extent or that there was more muscle co-activation during the production of the movements. Because passive joint viscoelastic properties and muscle co-activation were not included in the biomechanical model, it is possible that the net joint torque was less reliable from trial-to-trial during adduction. In addition, it is likely that the task was new to the participants. Consequently, it is possible that practice trials or more trials would be needed before participants develop consistent motor strategies that translate into greater reliability for adduction movements. Although results for adduction movements are less reliable than for other movements, the reliability is fair to good but caution should be taken as the 95% confidence interval is larger.

We assessed the impact of an estimated level of measurement error that would be acceptable in the calculation of the various parameters of the biomechanical model (i.e., length, moment of inertia and mass). The experimental standard errors of measurement for forearm and wrist power are larger than the predicted changes in forearm and wrist power due to simulated measurement errors. Therefore an acceptable level of accuracy would be if errors are kept within this boundary. As a result, measurement errors of ±5% should not impact forearm and wrist power measurements.

## Conclusions

In conclusion, the biomechanical model is reliable and robust to small measurement errors and could be used to assess the effectiveness of rehabilitation programs, to document the progression of athletes before, during and after specific exercise programs, and to conduct research-oriented testing of maximum wrist joint kinetics capacities. Future studies should aim at defining a set of normative values, for various age groups, for wrist joint torque and power in healthy and pathological individuals.

## Authors’ original submitted files for images

Below are the links to the authors’ original submitted files for images. Authors’ original file for figure 1 Authors’ original file for figure 2 Authors’ original file for figure 3
