# Supplementary figures and images for: Quantifying forearm and wrist joint power during unconstrained movements in healthy individuals
Source: J Neuroeng Rehabil. 2014 Nov 17;11:157. doi: 10.1186/1743-0003-11-157 (PMC4237781; doi:10.1186/1743-0003-11-157)

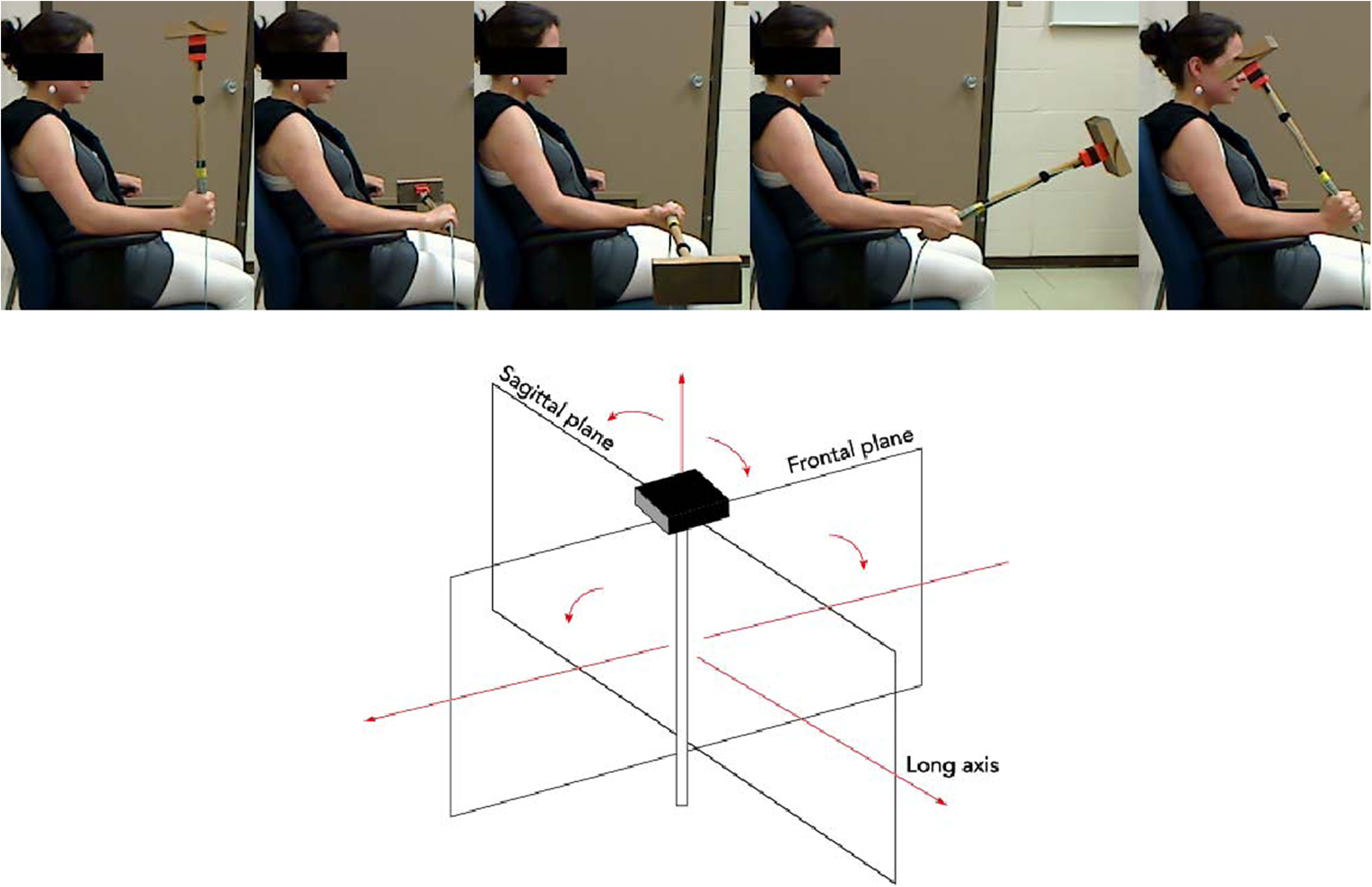

Supplement: Supplementary file 1 — Authors’ original file for figure 1 [file 12984_2014_669_MOESM1_ESM.tif]

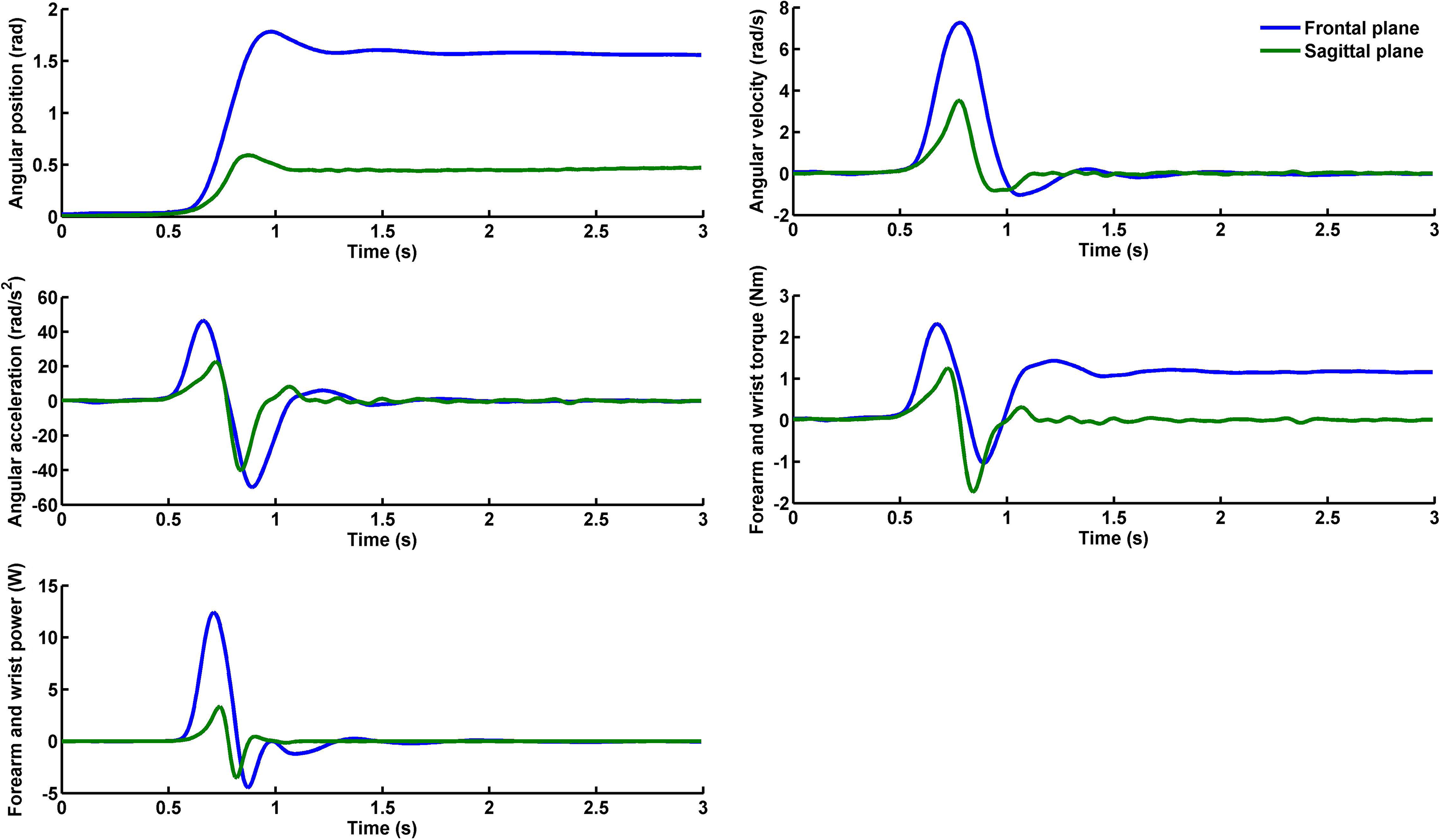

Supplement: Supplementary file 2 — Authors’ original file for figure 2 [file 12984_2014_669_MOESM2_ESM.tif]

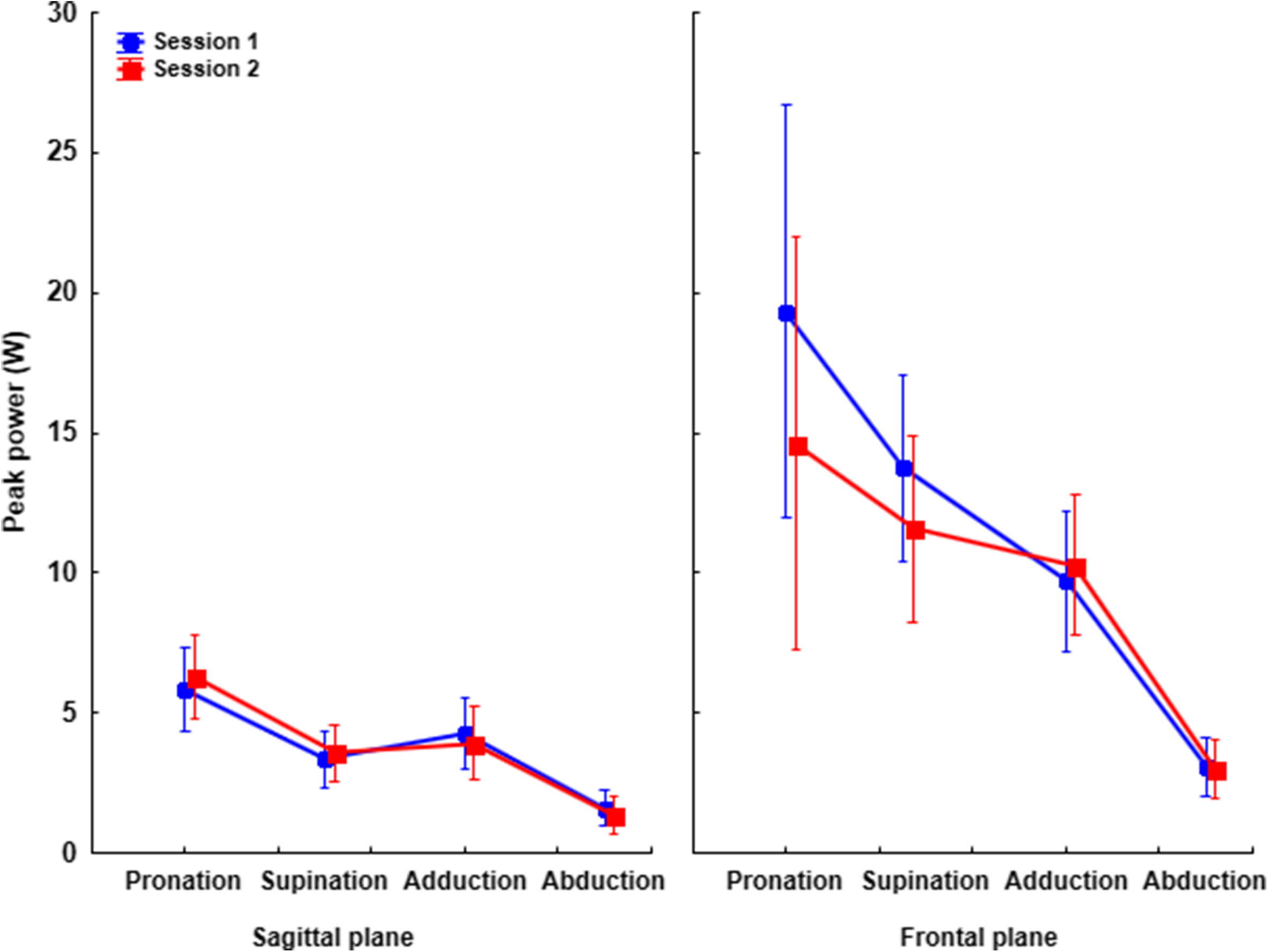

Supplement: Supplementary file 3 — Authors’ original file for figure 3 [file 12984_2014_669_MOESM3_ESM.tif]
